# Supplementary material for: The Dream and MEC NuRD Complexes reinforce SPR-5/MET-2 maternal reprogramming to maintain the germline-soma distinction
Source: bioRxiv. 2025 Jul 27:2025.07.23.666413. Preprint. [Version 1] doi: 10.1101/2025.07.23.666413 (PMC12330697; doi:10.1101/2025.07.23.666413)
Supplement: 1 [file NIHPP2025.07.23.666413V1-supplement-1.pdf]

bioRxiv preprint doi: https://doi.org/10.1101/2025.07.23.666413; this version posted July 27, 2025. The copyright holder for this preprint (which was not certified by peer review) is the author/funder, who has granted bioRxiv a license to display the preprint in perpetuity. It is made available under aCC-BY 4.0 International license.

Supplemental Figure 1

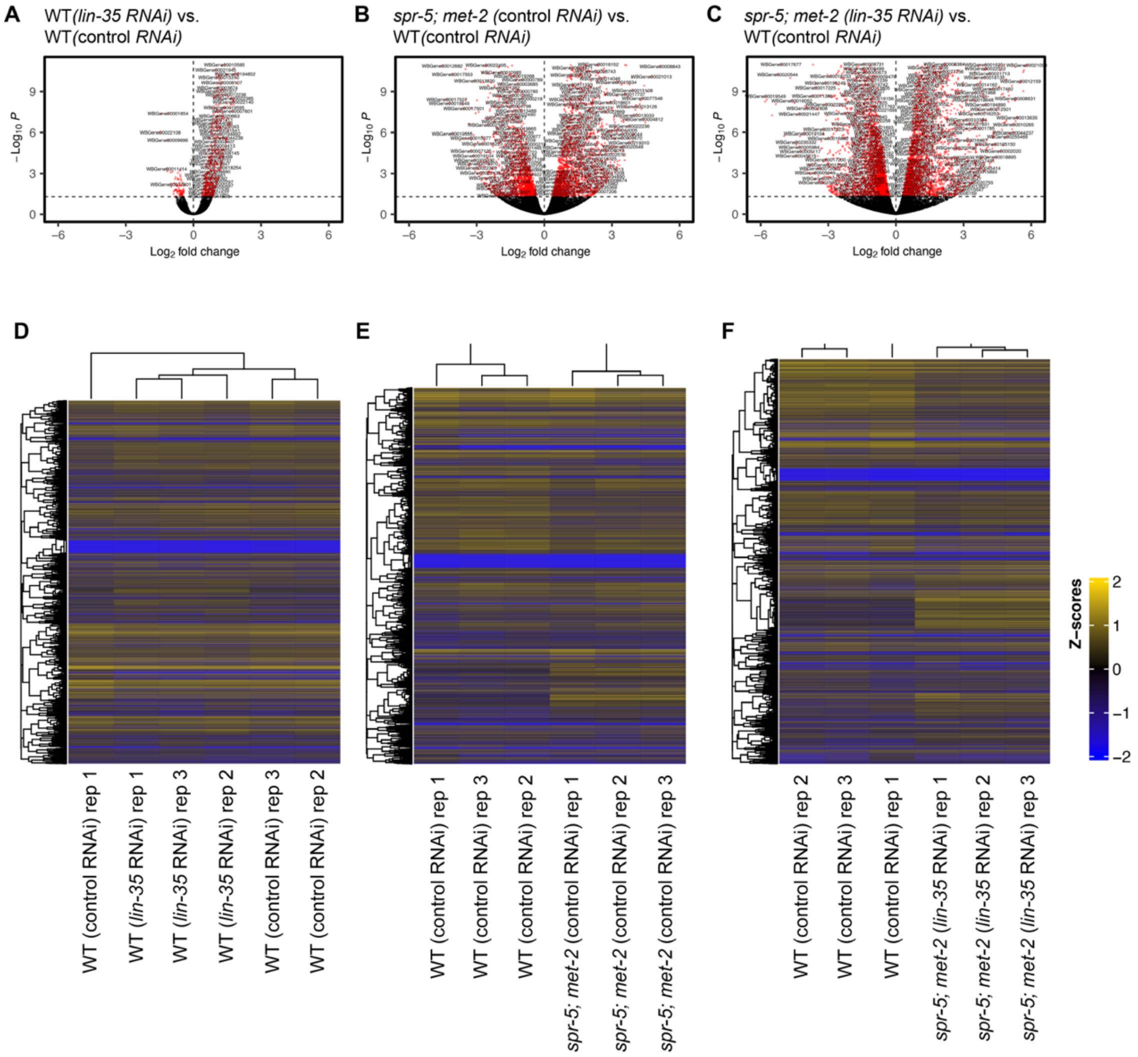

Supplemental Figure 1: **Differential expression and replicate comparison of RNAseq experiments performed on wild type, *spr-5; met-2* progeny fed either control or *lin-35* RNAi.** Volcano plot of log2 fold changes in gene expression (x-axis) by statistical significance (-Log_{10} P-value; y-axis) in L1 progeny of wild type (WT) hermaphrodites fed *lin-35* RNAi (A), *spr-5; met-2* hermaphrodites fed L4440 (control) RNAi (B), and *spr-5; met-2* hermaphrodites fed *lin-35* RNAi (C) compared to L1 progeny of wild type (WT) hermaphrodites fed control RNAi. Heatmap of differentially expressed RNA-seq transcripts between L1 progeny of wild type (WT) hermaphrodites fed control RNAi and wild type (WT) hermaphrodites fed *lin-35* RNAi (D), *spr-5; met-2* fed L4440 (control) RNAi (E), and *spr-5; met-2* fed *lin-35* RNAi (F). Data was scaled and hierarchical clustering was performed using complete linkage algorithm, with distance measured by calculating pairwise distance. Higher (blue) and lower (yellow) expression is reported as a z-score.

bioRxiv preprint doi: https://doi.org/10.1101/2025.07.23.666413; this version posted July 27, 2025. The copyright holder for this preprint (which was not certified by peer review) is the author/funder, who has granted bioRxiv a license to display the preprint in perpetuity. It is made available under aCC-BY 4.0 International license.

Supplemnetal Figure 2

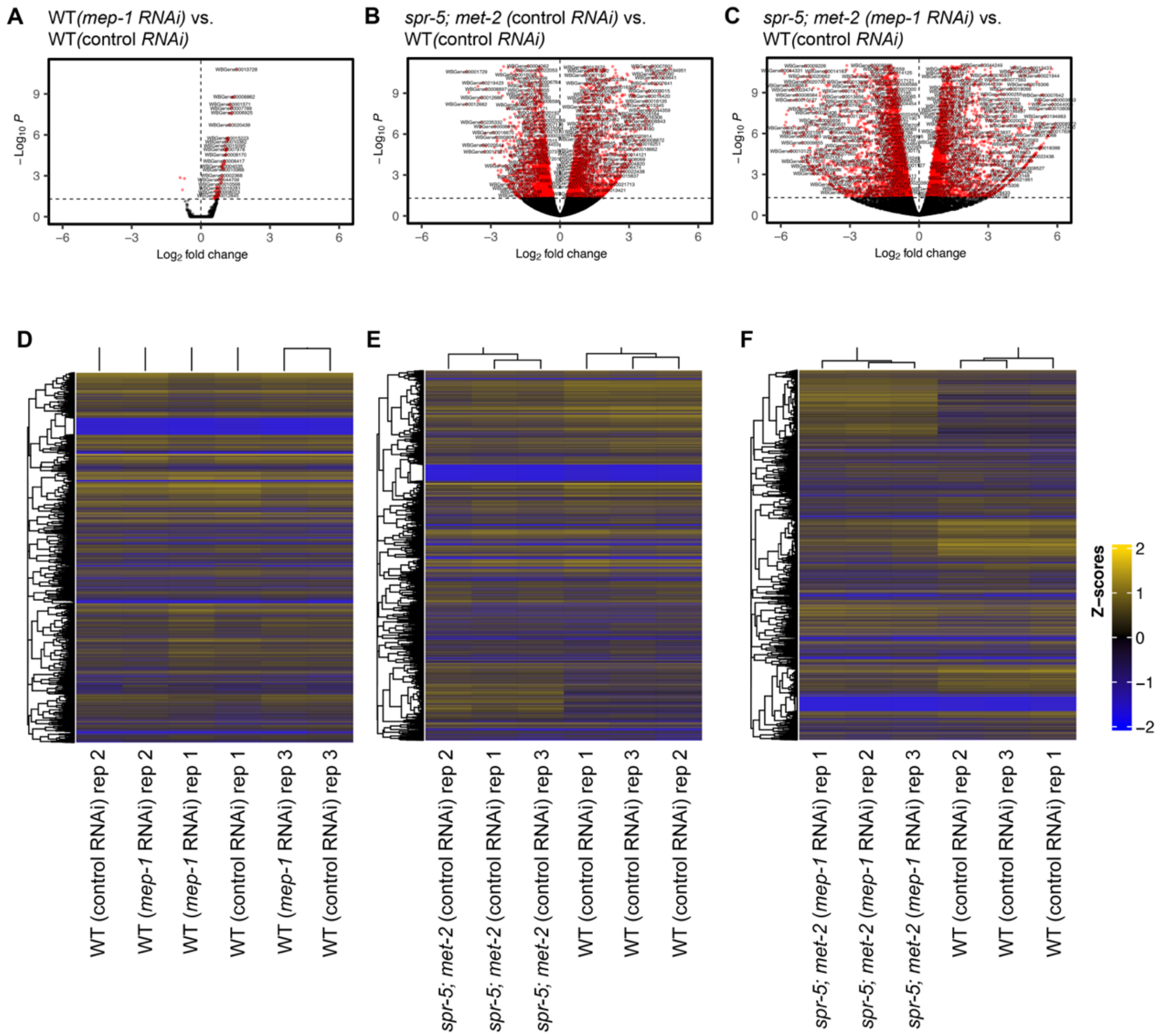

Supplemental Figure 2: **Differential expression and replicate comparison of RNAseq experiments performed on wild type, *spr-5; met-2* progeny fed either control or *mep-1* RNAi.** Volcano plot of log2 fold changes in gene expression (x-axis) by statistical significance (-Log_{10} P-value; y-axis) in L1 progeny of wild type (WT) hermaphrodites fed *mep-1* RNAi (A), *spr-5; met-2* hermaphrodites fed L4440 (control) RNAi (B), and *spr-5; met-2* hermaphrodites fed L4440 *mep-1* RNAi (C) compared to L1 progeny wild type (WT) hermaphrodites fed control RNAi. Heatmap of differentially expressed RNA-seq transcripts between L1 progeny of wild type (WT) hermaphrodites fed control RNAi and wild type (WT) hermaphrodites fed *mep-1* RNAi (D), *spr-5; met-2* fed L4440 (control) RNAi (E), and *spr-5; met-2* fed *mep-1* RNAi (F). Data was scaled and hierarchical clustering was performed

bioRxiv preprint doi: https://doi.org/10.1101/2025.07.23.666413; this version posted July 27, 2025. The copyright holder for this preprint (which was not certified by peer review) is the author/funder, who has granted bioRxiv a license to display the preprint in perpetuity. It is made available under aCC-BY 4.0 International license.

using complete linkage algorithm, with distance measured by calculating pairwise distance. Higher (blue) and lower (yellow) expression is reported as a z-score.

Supplemental Figure 3

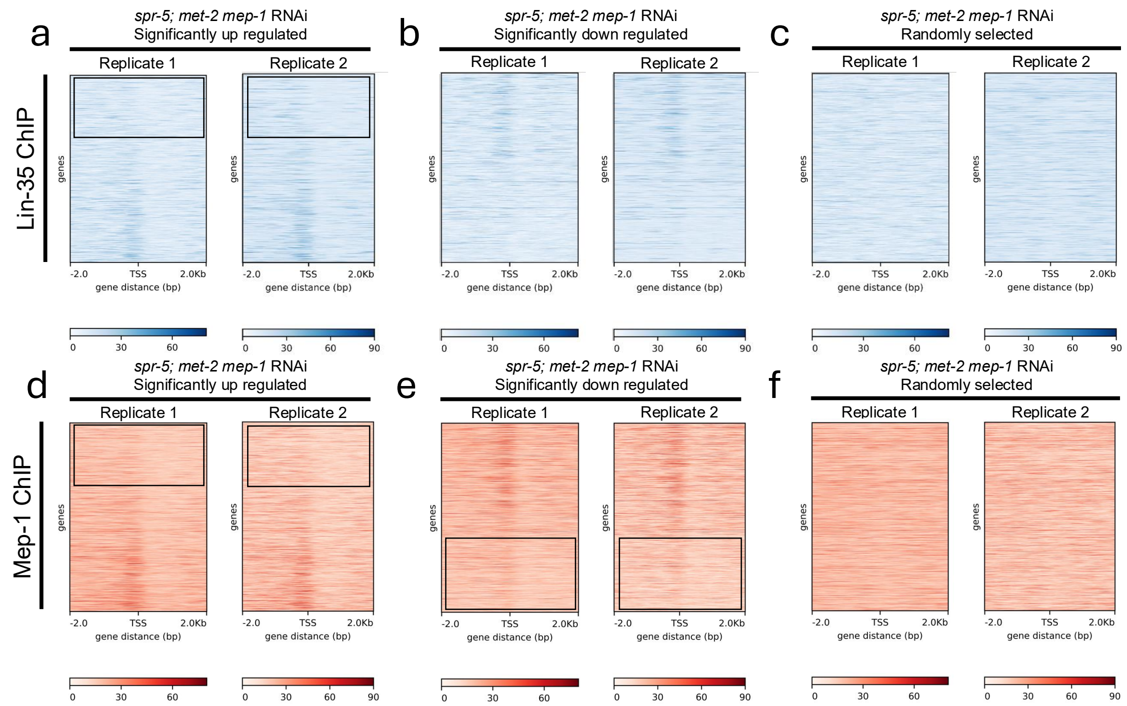

Supplemental Figure 3: Re-analysis of LIN-35 and MEP-1 ChIP-seq data performed on MEP-1 targets exaserbated in *spr-5; met-2* mutants. (A-F) Two replicate heat maps aligned from -2.0Kb to +2.0Kb with respect to the transcriptional start site (TSS) of LIN-35 (A-C) and MEP-1 binding (D-F) show a similar enrichment (darker blue or darker red in the heat maps) of binding around the TSS at genes that are significantly up regulated (A, D) upon knockdown of *mep-1* in *spr-5, met-2* mutants (from Fig. 4A), compared to no enrichment at an identical number of randomly chosen genes (C, F). The enrichment of LIN-35 binding is far less, but not completely gone at genes down regulated upon knockdown of *lin-35* in *spr-5, met-2* mutants (B) (from Fig. 4B), suggesting that LIN-35 acts predominantly as a repressor, but may ocassionally function as an activator. The enrichment of MEP-1 at genes down regulated upon knockdown of *lin-35* in *spr-5, met-2* mutants (from Fig. 4B) is reduced but not completely eliminated, suggesting that MEP-1 may sometimes function as an activator. In A-C the genes are listed from most highly up regulated at the top to least highly up regulated at the bottom. In D-F, the genes are listed from least highly down regulated at the top to most highly down regulated at the bottom. The boxes indicate the most highly up regulated (A, D) or down regulated genes (E) which do no bind LIN-35 (A) or MEP-1 (D, E).
